# Supplementary material for: Sustained Cognitive Improvement in Patients over 65 Two Years after Cochlear Implantation
Source: Brain Sci. 2023 Dec 3;13(12):1673. doi: 10.3390/brainsci13121673 (PMC10741742; doi:10.3390/brainsci13121673)
Supplement: Supplementary file 1 [file brainsci-13-01673-s001.zip › brainsci-2721485-supplementary.pdf]

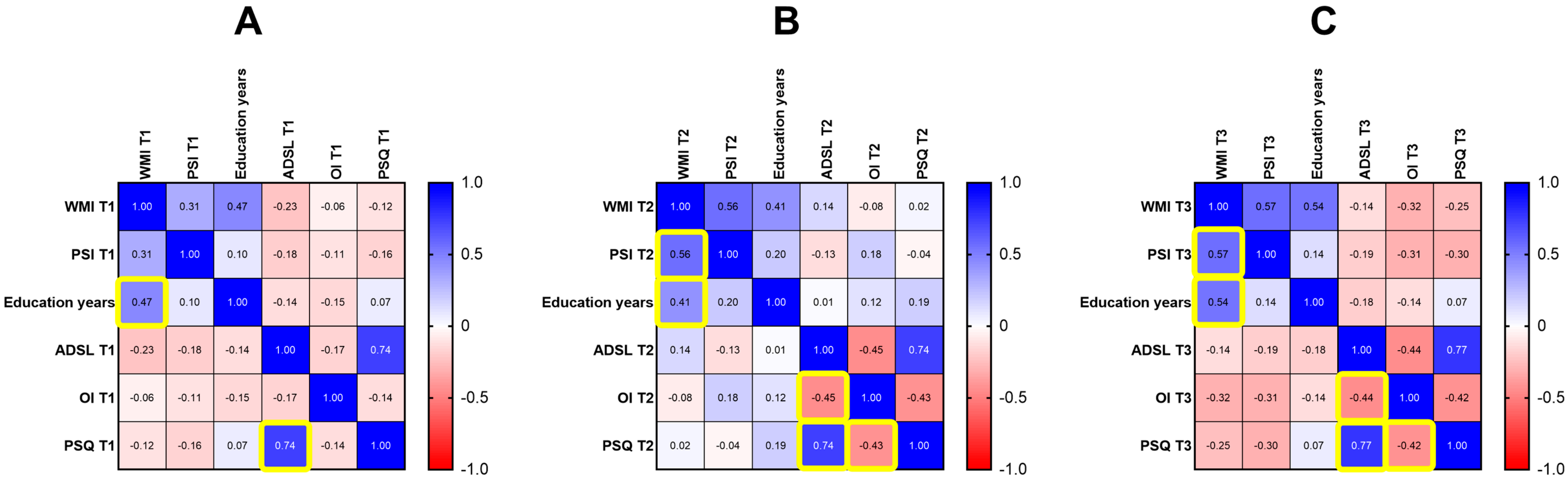

Supplementary Figure S1 Heat mapa depicting correlations between variables tested using Spearman's rank correlation coefficient at T1 (Figure 3A), T2 (Figure 3B), and T3 (Figure 3C). Negative correlations are shown in red, while positive ones are presented in blue. The significant correlations (p<0.05) are highlighted in yellow. WMI = Working Memory Index, PSI = Processing Speed Index, ADSL = General Depression Scale, OI = total score of Oldenburg Inventory, and PSQ = total score of Perceived Stress Questionnaire.
